# A Multiplier-Free Convolution Neural Network Hardware Accelerator for Real-Time Bearing Condition Detection of CNC Machinery

**DOI:** 10.3390/s23239437

**Published:** 2023-11-27

**Authors:** Yu-Pei Liang, Ming-You Hung, Ching-Che Chung

**Affiliations:** Department of Computer Science and Information Engineering, Advanced Institute of Manufacturing with High-Tech Innovations, National Chung Cheng University, Chia-Yi 621301, Taiwan; ypliang@cs.ccu.edu.tw (Y.-P.L.); honminyou@gmail.com (M.-Y.H.)

**Keywords:** fault diagnosis, convolution, neural networks, incremental network quantization, fixed-point arithmetic, real-time systems, field-programmable gate arrays, digital circuits

## Abstract

In various industrial domains, machinery plays a pivotal role, with bearing failure standing out as the most prevalent cause of malfunction, contributing to approximately 41% to 44% of all operational breakdowns. To address this issue, this research employs a lightweight neural network, boasting a mere 8.69 K parameters, tailored for implementation on an FPGA (field-programmable gate array). By integrating an incremental network quantization approach and fixed-point operation techniques, substantial memory savings amounting to 63.49% are realized compared to conventional 32-bit floating-point operations. Moreover, when executed on an FPGA, this work facilitates real-time bearing condition detection at an impressive rate of 48,000 samples per second while operating on a minimal power budget of just 342 mW. Remarkably, this system achieves an accuracy level of 95.12%, showcasing its effectiveness in predictive maintenance and the prevention of costly machinery failures.

## 1. Introduction

With the development of automated production, electric motors have been used in various industrial fields. Due to the different applications, the motor can be operated in harsh, fast, and overloaded environments. In this case, the motor parts may quickly fail, and the failure of the parts can cause considerable losses and result in safety risks for the operator. Therefore, fault diagnosis technology is one of the most important techniques in the modern industrial field. The fault types of motors and drive systems can be divided into stator faults, electrical faults of the rotor, and mechanical faults of the rotor [1], whereby bearing faults are the most common causes. Bearing faults account for 41% to 44% of all faults [2,3,4,5], and bearing faults cause noise, vibrations, and even system failures. If the fault can be diagnosed in time, then the loss due to the faults can be reduced. Also, the reliability and safety of the machine can be improved. The main fault conditions of the bearing include ball faults, outer race faults, and inner race faults. 

Currently, the bearing fault diagnosis methods can be divided into model-driven methods [6] and data-driven methods [7]. The model-driven approach must understand its physical principles and construct mathematical models accordingly. The data-driven method can be divided into four steps to diagnose bearing fault types: data acquisition, feature extraction, feature selection, and classification [8]. Also, the fault model can be built from the signals detected by the accelerometers. When dealing with non-linear and complex systems, the data-driven approach is a better choice because these systems are difficult to describe with accurate mathematics. More specifically, this paper adopts the data-driven approach and uses bearing fault vibration data provided by Case Western Reserve University (CWRU) [9].

Among various data-driven-based detection technologies, machine-learning-based methods, especially neural networks, have become popular because of their accuracy and adaptability. The burgeoning interest in this field has led to a recognition of the influence of uncertainty in the outcomes predicted by deep learning techniques, as highlighted in previous studies. For example, Liang et al. [10] tackled the issue of uncertainty and introduced a method based on the Gaussian process classifier (GPC), which is deeply anchored in Bayesian inference principles. Conversely, there exists a notable scarcity of publicly available data on real-world bearing faults, accompanied by challenges of data imbalance. To enrich training datasets and boost the precision of machine learning, generative adversarial network (GAN) technology [11,12] has been utilized to create new, analogous training samples, thereby crafting a highly refined model for identifying bearing faults. Beyond GAN, the use of transfer network technology has been embraced to overcome the hurdles in data gathering for the data-driven diagnosis of bearing faults, as indicated in [13,14].

However, the large computing load of machine learning and the complex data-preprocessing process may lead to fault detection not meeting the real-time requirement, and the faults may not be detected in time. Fortunately, implementing a machine learning model into a specific hardware accelerator can accelerate the computing speed. However, when a neural network is implemented in hardware, a large number of storage devices are required to store the parameters and calculation results of the neural network, and it may lead to the chip area being too large to be attached to CNC machines. Most hardware resources are located on the storage device instead of the calculation units. Therefore, memory reduction has become one of the foremost research topics. Approaches to memory reduction can be divided into network pruning [15,16] and network parameter quantization [17,18,19].

Weight pruning is a common technique for creating sparse networks that can eliminate unimportant weights during training to reduce memory space. In [15], the pruning of the neural network is divided into three steps. The first step is to train the network and distinguish the importance of the weights. The weight below the threshold is adjusted in the second step. The final step is to retrain the network. Retraining the network can seriously impact accuracy, but retraining can take a significant amount of time. On the other hand, the quantization method can be divided into two methods: weighting quantization and activation quantization, where the same effect can be achieved by using fewer bits. In [17], the weighting and activation are quantized into two values, and one bit can represent 1 and −1. Memory space can have a 32× reduction after the quantized network.

Additionally, the quantization method offers benefits beyond just memory reduction, extending to computational improvements. For instance, within a computing system, the prevalent multiplication operations in the convolutional layers of a neural network can be substituted with addition operations. This is achievable by quantizing the weights to power of two values. When this method is applied to hardware, the on-chip multipliers can be replaced with adders, leading to a substantial decrease in both power consumption and chip size. This efficiency gain stems from the fact that an adder, compared to a multiplier, occupies less chip area and is more energy-efficient. For example, in [18], the weighting has been quantized to three values (−1, 0, 1), and each weight needs to be represented by two bits. The memory space compression ratio can be more than 16 times, and the accuracy of this network is higher than that of a binary neural network. However, binary and ternary neural networks are easily affected by noise. In [19], a quantization method for incremental network quantization (INQ) is proposed. Initially, 50% of the total weights are quantized. Subsequently, the unquantized weights are retrained to compensate for the loss of accuracy. Then, half of the unquantized weights are quantized, and the same retraining process is repeated until all weights are quantized. Notably, in this work [19], weights are all quantized to the power of two. Then, the multiplication between the input and the weights can be replaced by shifting instead of multiplying. 

Although many existing excellent works discussed the weight pruning and quantization process, their usage for bearing fault detection hardware implementation gained little attention. On the other hand, despite that the computing time can be sped up by implementing neural network model to hardware, and that memory usage can be reduced by the technology of weight pruning and quantization, the complex data-preprocessing process still significantly influences the fault-detecting time. Therefore, reducing the complexity of the data-preprocessing process is also an important goal of this paper. More specifically, the main goal of this work is to propose a hardware solution for the accurate bearing fault diagnosis. In addition, the proposed method will achieve real-time processing and minimize the hardware resources.

The main contribution of this work can be summarized as follows.

1. In this work, a CNN-based hardware accelerator has been implemented for bearing faults, which can achieve real-time processing in industrial environments.
2. An improved incremental network quantization (INQ) method has been proposed to reduce the memory usage of the proposed hardware accelerator.
3. The complex multiplier operations have been removed from the proposed method to realize a multiplier-free accelerator.
4. The power consumption of the proposed accelerator can be reduced to 342 mW with a 140 MHz clock frequency.
5. The accuracy of the proposed accelerator can achieve 95.12% with only 8.69 K parameters.

The organization of the rest of the paper can be summarized as follows. Various of different type fault detection methods will be explored in Section 2. Next, the proposed method will be introduced in Section 3. More specifically, Section 3.1 describes the software architecture of our proposed model, and Section 3.2 provides the details of hardware implementation. The experimental results are then shown in Section 4. Finally, Section 5 will conclude this work.

## 2. Exploring Different Fault Diagnosis Methods

In this section, various machine-learning-based methods for fault diagnosis will be discussed to explore the research background.

(1) K-Nearest Neighbor (K-NN)

K-NN is an algorithm for classification and regression. The output is a classified ethnic group. The majority vote of the neighbors classifies the unclassified data. K indicates how many similar training data were selected. In [20], 30 features are extracted from the vibration signal, 24 of which are standard features for diagnosing rotating machine faults, and 6 are extracted by electromyography (EMG). Relief algorithm, Chi-squared, and information gain rank thirty features. The top 10 features are selected and fed into K-NN and random forest for classification, using five different vibration datasets for testing. The experiment results show that the features extracted by EMG are useful for diagnosing faults on rotating machines.

(2) Support Vector Machine (SVM)

The SVM is a supervised learning model that can find decision boundaries in the hyperplane to maximize both boundaries. When faced with non-linear responsibilities, kernel tricks can be added to map the original data in high-dimensional space. Many studies used SVM for bearing fault detection and achieved excellent results. In [21], a one-class v-SVM is used to detect abnormal vibration signals. If abnormal, a sensitivity test is used to select the fault frequency band through envelope analysis. The one-class v-SVM is highly input-dependent, and small changes to the input can result in reduced accuracy.

(3) Principal Component Analysis (PCA)

PCA is a method of statistical analysis and simplification of data. In [22], a solution for the unknown signal is proposed. The self-organizing map and principal component analysis (SOM-PCA) method analyzes the residual signal of the unknown signal. After the characteristic frequency of the faulty component has been separated, the SOM model classifies the four bearing states (normal condition, infield fault, field fault, and ball fault). This method can effectively distinguish the bearing condition.

(4) Neural Network (NN)

In [23], the feedforward multi-layer perceptron (MLP) neural network consists of three layers: an input layer, a hidden layer, and an output layer. After using the Laplace wavelet transform and genetic algorithm to preprocess the bearing vibration signal, the time and frequency domain features are extracted as inputs for the DNN. The features are the root mean square, standard deviation, kurtosis, wavelet spectrum frequency peak to the shaft rotational frequency ratio, and wavelet power spectrum maximum amplitude to the overall amplitude ratio. The genetic algorithm aims to reduce the number of hidden-layer nodes to improve the classification speed. Finally, the bearing health status is classified into four bearing categories. The Laplace wavelet transform is very sensitive to noise, and when it encounters a signal with a sizeable periodic component, it is easy to generate errors. In [24], the vibration signal characteristics are extracted from the frequency domain to reduce the size of the data and then fed into the DNN model for fault classification. This approach reduces the network architecture and shortens the calculation time. The Fourier transform is useful for smoothing signals and may not reflect features in non-uniform signals. 

Moreover, in [25], a quantum-inspired differential evolution (MSIQDE) algorithm is proposed based on the improvement of the multi-strategy to optimize the DBN connection weights and improve accuracy. Moreover, the CNN-based method has become mainstream for fault diagnosis technology because of its high accuracy. In [26], the discrete wavelet transform (DWT) splits the original signal into different scales, uses CNN for feature extraction, and finally performs fault classification using the softmax classifier, which performs better than other wavelet-based methods. However, the input is the four components of the signal. The parameters of the first layer are 32 × 32 × 4 × 32, and there are many parameters. Therefore, the chip area of hardware circuits and power consumption are unfavorable. In [27], the original signal is randomly sampled, and the input signal is converted into a two-dimensional image and then fed to the LeNet-5 CNN for classification. The accuracy of the three experimental conditions is more than 99%.

More recently, the study cited as [28] introduced a CNN model specifically developed for detecting bearing faults, utilizing both vibration and acoustic signals. In this study, the short-time Fourier transform (STFT) method was applied to transform these signals into a time frequency representation. The findings from this research showed that the proposed model achieved notably high accuracy in classification tasks. In contrast, the study referred to as [29] went beyond signals produced by CNC machines (like vibration, current, and acoustic signals) and introduced an innovative approach to fault detection. This approach involved the use of event-based cameras and vision sensing techniques to develop a contactless fault detection method. The results of their experiments showed high accuracy under specific conditions, also emphasizing that the effectiveness of this camera-based solution is greatly influenced by lighting conditions.

However, to enhance the accuracy, many existing works tend to select complex preprocessing methods, taking several important features and then using neural networks to classify them. Complex pretreatment will greatly burden hardware implementation, so this paper uses the method of [27]. The pretreatment stage is relatively simple, and the accuracy is not worse than other methods.

## 3. The Proposed Methodology

In this research, we focus on developing a real-time hardware solution to identify faults in CNC machinery. Our approach employs a CNN as the predictive mechanism for fault detection. The CNN model primarily uses images as input, which, in our case, are derived from sensors attached to CNC machines. The essence of real-time processing in our project is the system’s ability to complete the fault prediction before the sensors produce a subsequent image. For example, if an input image is made up of 4096 data points and the sensor outputs every × nanoseconds, then the model’s prediction time per image should be less than 4096× nanoseconds. In simpler terms, the model must process each image swiftly to prevent delay accumulation, thereby maintaining real-time efficiency. To achieve this, we simplify complex data preprocessing steps in our model. Besides streamlining preprocessing, reducing the computational delay in the CNN is essential, particularly in processes like convolution. Typically, a CNN with more parameters yields higher accuracy but also longer prediction times. Therefore, balancing the CNN’s architecture size and its accuracy is crucial. On the hardware side, we prioritize efficiency; adders are used instead of multipliers to decrease computation time, power consumption, and chip area in our design. Moreover, real-time performance, accuracy, and hardware cost are our prime considerations. We have endeavored to reduce memory usage and hardware overhead by minimizing the bits needed for data representation. However, this reduction can significantly affect prediction accuracy. This paper will also explore the trade-off between accuracy and hardware cost. Detailed explanations of our methodology will be discussed in the following subsections.

### 3.1. Software Design

#### 3.1.1. Dataset Preprocessing

First of all, we reduce the data preprocessing process, and this section will introduce the details of the dataset we use in this paper and the dataset preprocessing process. As aforementioned, in this work, we use the CWRU [9] dataset to train a CNN model to detect the bearing faults. The test equipment at CWRU includes a two-horsepower (HP) motor, torque sensor and encoder, a force meter, and control electronics. Svenska Kullagerfabriken (SKF) (Gothenburg, Sweden) bearings and NTN equivalent bearings are used as test bearings to support the motor shaft. The test bearings generate a single point of failure by discharge processing. SKF bearings produce 0.007-, 0.014-, and 0.021-inch single-point failures, and NTN equivalent bearings produce 0.028- and 0.04-inch single-point failures.

The defective bearing is installed in the electric motor, and the electric motor runs at a constant speed. Each motor loads 0–3 HP (approx. 1730–1797 r.p.m). Bearing vibration data are collected by three accelerometers mounted on the motor support substrate, the drive-end motor housing, and the fan-end motor housing. Subsequently, the collected vibration signals are processed in MATLAB. Each file contains multiple accelerometer data. Moreover, there are four bearing datasets in the CWRU datasets: normal reference, 12 K drive bearing failure, 48 K drive bearing failure, and 12 K fan-end bearing failure. There are three types of fault data: inner race, ball fault, and outer race. The position of the outer race fault concerning the bearing load area directly affects the vibration response, so the outer race fault data contain data at three different locations: three o’clock position (orthogonal), six o’clock position (center), and twelve o’clock position (opposite).

The vibration dataset is used to train the CNN model in this work. More specifically, the vibration signals are converted into images sequentially. The bearing status is classified into ten categories according to the diameter of damage and type of fault, and if the vibration signal can be classified into one of ten categories, then the CNN network outputs the appropriate category. The label and corresponding bearing status are shown in Table 1. The CWRU bearing vibration dataset for bearing fault detection consists of a total of three accelerometer data, respectively: drive-end accelerometer data (DE), fan-end accelerometer data (FE), and basic accelerometer data (BA). In this paper, data from fan-end (FE) accelerometers are used.

To convert a vibration signal to a gray-scale image through normalization, the normalization requires a time domain signal’s maximum and minimum values and performing multiplication and division operations, as shown in Equation (1). When implementing CNN hardware, it takes 4096 cycles to find the minimum and maximum values of the vibration signal. The preprocessing of the vibration signal will take a long time and require memory and computing resources to process. Therefore, this paper directly arranges the values of vibration signals into 64 × 64 images.

(1) $$y_{i}=\frac{{(x}_{i}-min)}{\left(max-min\right)}\times 255$$

Note that, in order to avoid waiting time, this study does not make a normalization for the vibration signal. The vibration signal is converted directly as an image, and each image size is 64 × 64. All images are divided into training images in the proportion of 64:16:20, verified, and tested. There are 8306 images, of which 5317 are training images, 1329 are validated images, and 1660 are test images. Training, validation, and test images are randomly selected from all images, not from each category. Therefore, the number of training, validation, and test images in each category is not exactly 64:16:20. The number of training, validation, and test images in each category is shown in Table 2.

#### 3.1.2. Architecture of the Proposed CNN Model

**Figure 1:**
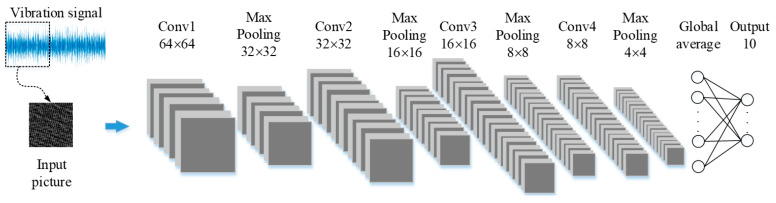
The proposed CNN network architecture.

The proposed CNN network architecture for bearing condition detection is shown in Figure 1. After arranging the vibration signals into images, the bearing conditions are classified using the proposed CNN network. The proposed CNN architecture includes the convolutional layers (Conv1 to Conv4), the max pooling layers, the global average pooling layer, and the fully connected layer. If the global average pooling layer is used in the network instead of the convolution results being output directly to the fully connected (FC) layer, then the parameters of the neural network are greatly reduced, and overfitting is prevented. The Conv includes a zero-padding layer, a convolution layer, and an activation layer.

This research examines four kernel sizes: 1 × 3, 1 × 5, 2 × 2, and 3 × 3. The vibration signals are arranged on the image from the top to bottom and left to right. Notably, there is a lack of continuity among values from distinct data rows situated at the same point. To illustrate, the initial values of the first and second rows do not represent continuous signals. As a result, for the purposes of this research, kernel sizes of 1 × 3 and 1 × 5 are more suitable. For convolutional layers, Conv1 utilizes 8 filters, Conv2 employs 16 filters, while Conv3 and Conv4 use 32 filters to gauge accuracy and pinpoint the optimal kernel size. The outcomes of the simulations can be found in Table 3. With a 1 × 5 kernel size, we achieve an accuracy rate of 97.16%, leading to the adoption of this size for our study.

Eight distinct architectures are trained to ascertain the optimal number of layers and the number of filters within each layer. Out of these, half feature three convolutional layers, while the remaining half have four convolutional layers. All architectures employ a kernel size of 1 × 5 and leverage global average pooling, leading to outputs for ten classes. As illustrated in Table 4, the accuracy of using three convolutional layers is on par with that of four convolutional layers. However, the parameter count for the three-layer setup is not necessarily lower. For instance, Architecture 3, comprising three convolutional layers, has filters distributions of 32 in Conv1, 64 in Conv2, and 128 in Conv3. It achieves an accuracy rate of 97.34% with a parameter count of 13,530. In comparison, Architecture 6, with its four convolutional layers, allocates 8 filters to Conv1, 16 to Conv2, and 32 to Conv3 and Conv4. Despite its accuracy being marginally lower by 0.18% compared to Architecture 3, it requires 4840 fewer parameters. Based on this, our study selects Architecture 6.

Table 5 compares prior works focusing on CWRU bearing fault detection but employing varying network architectures and preprocessing techniques. This study takes into account hardware implementation; therefore, it adopts preprocessing methods that are easy to implement and aim to minimize the architecture to achieve parameter lightness. In Table 5, the network parameters of the proposed model are more than 30-fold fewer than other architectures. In contrast, the accuracy of the proposed model is only slightly lower by 2.54% compared to the others.

**Figure 2:**
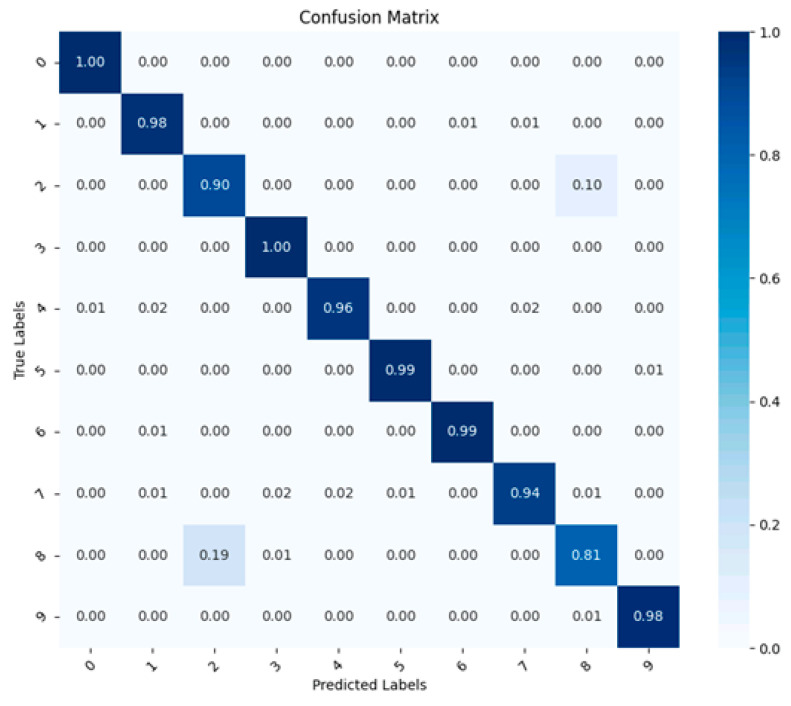
The confusion matrix of the proposed CNN network architecture.

To assess our trained model’s ability to classify accurately, we create a confusion matrix for the proposed CNN architecture, as shown in Figure 2. This matrix uses the x-axis to display predicted categories across ten classes and the y-axis to indicate the actual labels of the input images. Each value in the matrix reflects the likelihood of a specific outcome. For example, a value at the intersection of the actual label 0 and predicted label 0 indicates the probability that an image, truly belonging to class 0, is correctly predicted as class 0. High values along the diagonal of the matrix, nearing 1, suggest strong classification accuracy. Notably, all diagonal values being close to 1 in Figure 2 demonstrates that our CNN model has a high level of classification precision.

### 3.2. Hardware Implementation

Upon successfully training the CNN model in a software environment, our next step involves starting to implement this model into a hardware accelerator. This section delves into strategies for minimizing power consumption and chip area while enhancing computational speed. A significant factor in the circuit’s power consumption is attributed to off-chip memory access. Frequent read and write operations on the same memory device substantially limit the energy efficiency of the circuit. Consequently, quantizing the CNN network becomes imperative to lessen memory access time and power consumption, thereby boosting the speed of computations.

**Figure 3:**
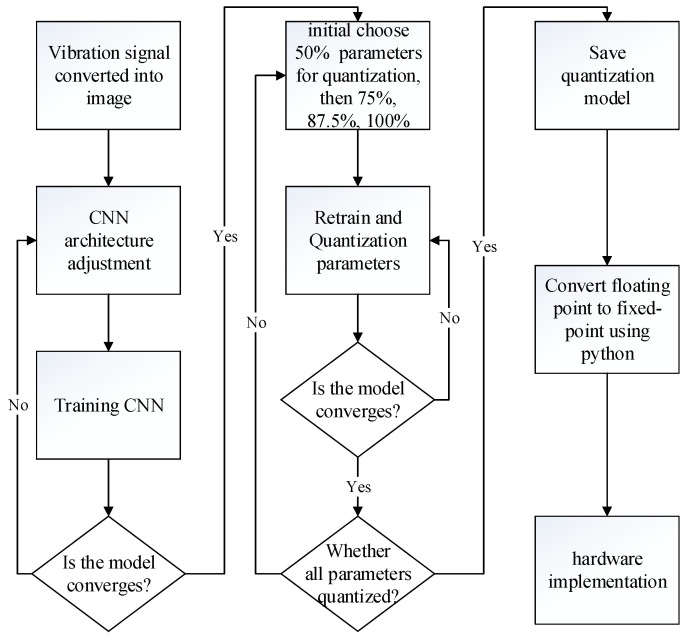
From software training to quantization, parameter extraction, final software verification, and hardware implementation.

The entire design process proceeds from training the CNN network model to the hardware implementation, as shown in Figure 3. The first step is to convert 4096 vibration signals per image into 64 × 64 images using the signal-to-image conversion method mentioned in [27]. The second step is to train the CNN network architecture and consider power consumption and computing speed in the hardware implementation. Therefore, the network structure is kept lightweight to reduce network complexity and the number of parameters, but high accuracy is still required.

The third step involves parameter quantization. This research draws inspiration from the incremental network quantization method [19] with further enhancements. In [19], the quantization process is divided into four stages, initially quantizing 50% of all model parameters, followed sequentially by 75%, 87.5%, and 100%. During the retraining process, ref. [19] utilizes unquantized parameters for adjustments to compensate for the accuracy loss caused by quantization. On the other hand, in this work, all parameters are used for retraining. Notably, this work does not employ pruning since its effects are insignificant.

In the fourth step, the values are represented in fixed-point format after quantizing all parameters. However, TensorFlow is used when training the proposed CNN, and floating-point computations are applied. Therefore, during hardware implementation, as fixed-point computations can lead to accuracy loss, it is essential to evaluate the proposed CNN in Python to assess the precision loss when choosing the minimum bit-width implementation. After confirming the precision loss and determining the bit-width, we proceed to write the Verilog register transfer level (RTL) code to implement the functionality of each component in the proposed CNN. Subsequently, we synthesize the code using Xilinx Vivado and obtain the implementation results. 

**Figure 4:**
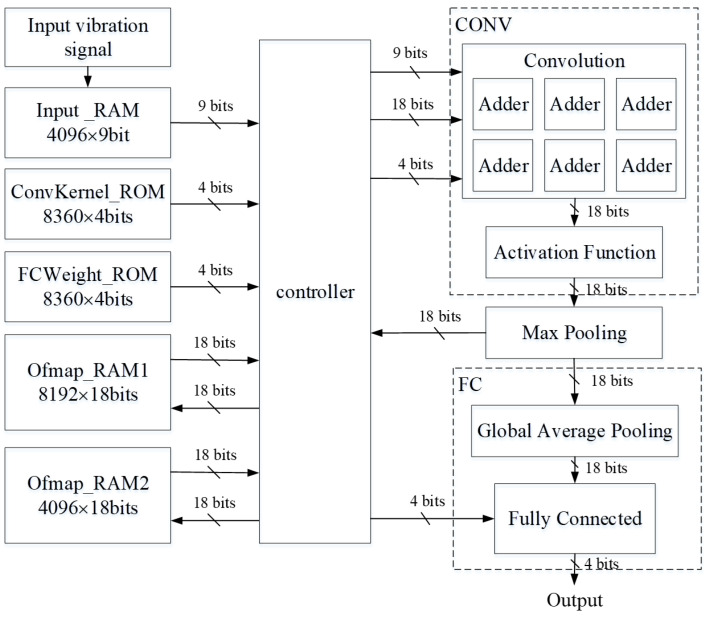
The proposed CNN network block diagram.

The proposed CNN network hardware block diagram is shown in Figure 4. The entire calculation process can be divided into three blocks: Conv, max pooling, and FC. The Conv is responsible for the zero-padding, dynamic fixed-point adjustment, convolution calculations, and activation function. The image size output after the convolution operation is smaller, so the input image needs to be zero-padded to maintain the same input and output image size. Dynamic fixed-point adjustments are used in the architecture, and each layer’s integer and decimal bits are different. In this study, the whole number of integers and decimal places is adjusted in the Conv, and the entire convolution operation is carried out only after adjustment. The convolution contains six adders, five of which are used for the kernel calculation of the convolution operation, and one is for the partial sum accumulation. The activation function chooses to use ReLU because ReLU is very simple. The max pooling block is responsible for performing max pooling and controlling whether the output is stored to Ofmap_RAM, and when four layers of convolution are complete, the results are output to the FC block. The FC block is responsible for global average pooling and fully connected operations and compares ten output values, outputting the maximum label. There are four convolution layers and four max pooling layers in the proposed CNN network. The max pooling results from the first to the third layer are transmitted to Ofmap_RAM1 or Ofmap_RAM2, and the max pooling results in the fourth layer are transferred to the FC block for operations. The global average operation and the fully connected layer operation are performed in the FC block. After four maximum pooling layers, only 4 × 4 images remain in the output. Therefore, the global average operation can be calculated by shifting four bits to the right instead of division. When the fully connected layer is calculated, the label with the maximum value is output. ConvKernel_ROM only stores the weight of the convolution layer. The weight of the fully connected layer is stored in the FCWeight_ROM, which makes it more convenient to take the value when performing fully connected layer operations. The memory management method of the ping-pong architecture consists of two memory banks, where one memory provides the value, and the other memory stores the result of the operations. Since the CNN network proposed in this design has the most significant output values on the first and second layers, the number of first- and second-layer output values determines the memory size.

The quantization process is divided into four stages, initially quantizing 50% of all model parameters, followed sequentially by 75%, 87.5%, and 100%. The quantization process is shown in Algorithm 1. First, random numbers produce a quantization order of parameters for each layer and quantify the parameters of that layer based on the quantization order and the quantization phase. The model is quantified once it documents the accuracy of the quantization, and then the model, maximum precision, and quantization order to be trained and quantized is entered into Algorithm 2 for retraining and quantization. If the accuracy of the output of Algorithm 2 is not higher than before, then the quantization method is replaced. If all three quantization methods are already in use, then the next phase of quantization will take place.

This study employs three distinct methods in the quantization process, as detailed in Algorithm 2. The first method involves quantizing after each training epoch. The second method quantifies after each training batch. Meanwhile, the third approach activates quantization once the verification accuracy surpasses a predetermined threshold. If the conditions for quantization are met, then the quantization model and the quantization order are transmitted to Algorithm 3. If the quantization accuracy surpasses the peak accuracy, then the model will be preserved. While all three methods are applied, the first and third methods often yield higher training accuracies at the expense of lower verification accuracies. Conversely, the second method effectively narrows the training and verification accuracy gap.
**Algorithm 1.** Select order and training$\mathrm{INPUT}:\;\mathrm{trained}\_\mathrm{network}\;\mathrm{Net}\;\mathrm{OUTPUT}:\;\mathrm{quantized}\_\mathrm{network}\;\hat{Net}$ $\;//\mathrm{LW}\_\mathrm{NUM}[n]\;:\;\mathrm{number}\;\mathrm{of}\;\mathrm{layer}\;n\;\mathrm{weights}\;\hat{Net}=\mathrm{Net};$ $\mathrm{for}\;i\;\mathrm{in}\;\mathrm{range}\;(\max(n)):\;\;\;\mathrm{order}[i]=\mathrm{RandomOrder}(\mathrm{LW}\_\mathrm{NUM}\;[i])\;\mathrm{for}\;m\;\mathrm{in}\;\mathrm{range}([0.5,\;0.75,\;0.875,\;1])\;\;\;\mathrm{Qmethod}=0;\;\;\;\max\_\mathrm{acc}=0;\;\;\;\mathrm{original}\_\mathrm{acc}=0;\;\;\;\mathrm{for}\;i\;\mathrm{in}\;\mathrm{range}(\max(n)):\;\;\;\;\;\mathrm{orderM}=\mathrm{order}[i][0:\;\mathrm{LW}\_\mathrm{NUM}\;[i]*m]\;\;\;\hat{Net}=\mathrm{QuantizationNetwork}(\hat{Net},\;\mathrm{orderM});$ $\;\;\mathrm{while}(\mathrm{Modelnotconvergent})\;\;\;\;\;\mathrm{original}\_\mathrm{acc}=\max\_\mathrm{acc};\;\;\;\;\;\mathrm{while}(\mathrm{Modelnotconvergent})\;\;\;\;\;\;\;\hat{Net}=\mathrm{QuantizationMethod}(\hat{Net},\;\mathrm{orderM},\max\_\mathrm{acc},\mathrm{Qmethod})$ $\;\;\;\;\mathrm{if}\;(\mathrm{original}\_\mathrm{acc}==\max\_\mathrm{acc})\;\;\;\;\;\;\;\mathrm{Qmethod}=\mathrm{Qmethod}+1;\;\;\;\;\;\;\;\mathrm{if}\;(\mathrm{Qmethod}==3)\;\;\;\;\;\;\;\;\;\mathrm{Qmethod}=0;\;\mathrm{return}(\hat{Net})$

**Algorithm 2.** Quantization method$\mathrm{Input}:\;\mathrm{Net}\;,\;\mathrm{orderM}\;,\;\max\_\mathrm{acc}\;,\;\mathrm{Qmethod}\;\mathrm{Output}:\;\mathrm{quantized}\_\mathrm{network}\;\hat{Net}$ $\;\mathrm{if}\;(\mathrm{Qmethod}==0)\;\;\;\hat{Net}=\mathrm{TrainingNetwork}(\mathrm{Net});$ $\;\;\hat{Net}=\mathrm{QuantizationNetwork}(\hat{Net},\;\mathrm{orderM});$ $\mathrm{else}\;\mathrm{if}\;(\mathrm{Qmethod}==1)\;\;\;\mathrm{while}\;(\mathrm{AnEpochisnotComplete}.)\;\;\;\;\;\;\hat{Net}=\mathrm{TrainingNetworkOneBatch}(\mathrm{Net});$ $\;\;\;\;\hat{Net}=\mathrm{QuantizationNetwork}(\hat{Net},\;\mathrm{orderM});$ $\mathrm{else}\;\;\;\hat{Net}=\mathrm{TrainingNetwork}(\mathrm{Net});$    **if** (validation_acc >= SettheAccuracyRate) $\;\;\;\;\hat{Net}=\mathrm{QuantizationNetwork}(\hat{Net},\;\mathrm{orderM})$; $\mathrm{validation}\_\mathrm{accuracy}=\mathrm{TestNetwork}(\hat{Net})$ $\mathrm{if}\;(\mathrm{validation}\_\mathrm{acc}\;>\;\max\_\mathrm{acc})\;\;\;\max\_\mathrm{acc}=\mathrm{validation}\_\mathrm{acc};\;\;\;\mathrm{SaveModel}(\hat{Net});$ $\;\;\mathrm{return}\;(\hat{Net})$; **else**
     return (Net);

Parameters are quantized to the nearest value among the 15 options. These values are 1, −1, 0.5, −0.5, 0.25, −0.25, 0.125, −0.125, 0.0625, −0.0625, 0.03125, −0.03125, 0.015625, −0.015625, and 0, as shown in Algorithm 3. After quantization, the computational procedure does not use multiplication; only shifting the operation is required.
**Algorithm 3.** Quantization network$\mathrm{Input}:\;\mathrm{Net}\;,\;\mathrm{orderM}\;\mathrm{Output}:\;\mathrm{quantized}\_\mathrm{network}\;\hat{Net}$ $\;\mathrm{Target}=\{0,\pm 2^{0},\pm 2^{-1},\pm 2^{-2},\pm 2^{-3},\pm 2^{-4},\pm 2^{-5},\pm 2^{-6}\}$ $\mathrm{for}\;i\;\mathrm{in}\;\mathrm{range}\;(\mathrm{number}\;\mathrm{of}\;\mathrm{Net}\;\mathrm{layers})\;\;\;\mathrm{for}\;j\;\mathrm{in}\;\mathrm{range}(\mathrm{orderM})\;\;\;\hat{Net}[i][j]=\mathrm{QuantizeasApproximateValue}\;(\mathrm{Target},\;\mathrm{Net}[i][j])$ $\mathrm{return}(\hat{Net})$

In the proposed CNN model, there are 8680 parameters, excluding biases. Throughout the training process, 32-bit floating-point numbers facilitate the calculations of the neural network model. Without quantization, storing all these parameters necessitates 277,760 bits of memory. Models that are neither compressed nor quantized lead to increased chip area and greater power demands. Consequently, we employ the improved incremental network quantization method to quantize the parameters, with each parameter being represented using just four bits. This results in savings of 243,040 bits at the cost of losing 1.62% accuracy. Table 6 exhibits the accuracy achieved at varying quantization levels. The remaining unquantized parameters can counteract the losses from quantization. Therefore, even after quantizing 87.5% of the parameters, the verification accuracy remains unchanged.

To demonstrate the reliability of our approach, we conduct a series of experiments for cross-validation analysis. In this experiment, we adopt a shuffle–split strategy that randomly samples elements from datasets in each iteration. More specifically, we resample our dataset ten times, generating unique training and testing sets labeled from v1 to v10. For each set, we retrain our model. The outcomes of these experiments are presented in Table 7. As observed in the table, our proposed network consistently achieves an accuracy rate above 91% in all ten instances of resampling.

**Figure 5:**
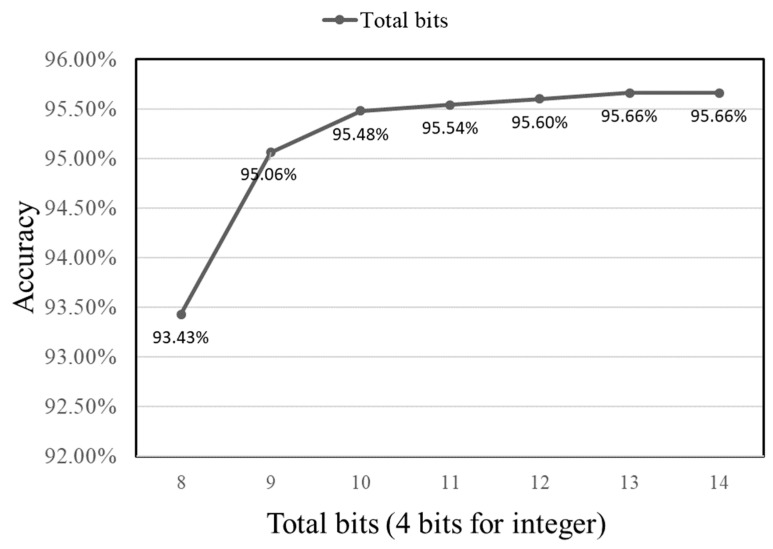
Accuracy vs. presenting bits of the input value.

Moreover, sensor data are continuously fed into the operational circuit in the hardware configuration. Consequently, a dedicated memory segment is essential to store these sensor data. The vibration signal’s minimum and maximum values are −7.1409 and 7.5856, respectively. As a result, one bit is allocated for the sign and three bits for the integer part. Next, we experiment with varying the precision from 4 to 10 decimal bits to assess accuracy implications. The outcomes of these tests can be found in Figure 5. Note that, in Figure 5, the x-axis denotes the total presenting bits, which includes the 4-bit integer of the input, and the y-axis indicates the accuracy. Assuming an input value is represented using a 32-bit floating point and an image contains 4096 values, the necessary memory storage amounts to at least 131,072 bits. When each value is denoted using four bits for the integer part and five for the fractional part, summing up to nine bits in total, there is a slight reduction in accuracy by 0.42%. However, this approach allows for significant memory savings of 94,208 bits.

During the execution of the CNN hardware circuit, the input is accessed multiple times. Hence, two instances of Ofmap_RAM are required to implement the ping-pong architecture. While one memory handles the data reading process, the other memory stores the results from the CNN operations. The outputs from each layer are also represented in fixed-point format to conserve space in Ofmap_RAM.

Owing to the vast variability in the output values across each layer of the neural network, it is crucial to assess the bit requirement for each layer before transitioning values from 32-bit floating-point to fixed-point format. The number of output bits is minimized by trimming extra bits before they are relayed to the subsequent layer for processing. It is worth noting that the count of output bits directly influences memory consumption. A Python-based simulation of the CNN network’s computation process is employed to quickly determine the integer and fractional bits that need to be retained for each layer’s output. During this Python-based CNN operation simulation, each layer’s maximal and minimal values are logged throughout the computation, with the results shown in Table 8.

This study examines two distinct fixed-point methods to calculate the necessary bit count. The first approach uses a consistent representation with a set number of integer and decimal bits per layer. Specifically, the integer part is set to 12 bits tested across decimal bits ranging from 5 to 10. The second method, dynamic fixed point, adapts the integer digits based on the layer’s maximum value. For example, Conv1 through FC have integer bits set at 5, 8, 10, 12, and 10, respectively, and are tested using 17 to 22 bits to evaluate any accuracy drop in the CNN.

**Figure 6:**
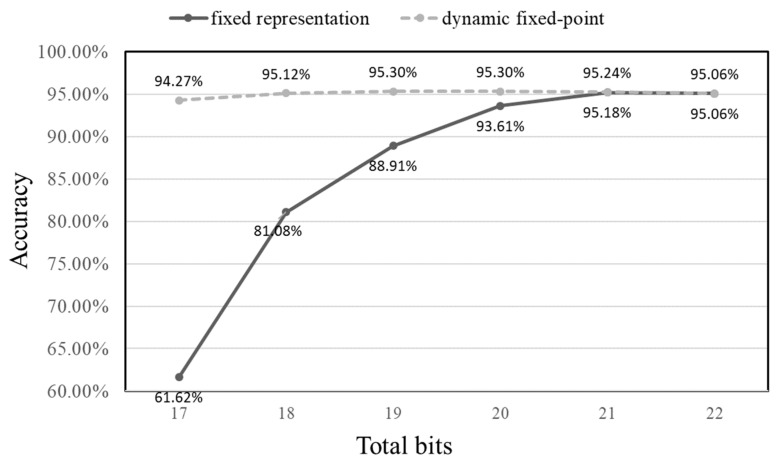
Effect on accuracy vs. bit number of the fixed representation and dynamic fixed point.

Results for both methods are shown in Figure 6. The data suggests that the dynamic fixed-point method outperforms the consistent representation, especially when fewer bits are employed. A clear observation from Figure 6 is that the dynamic approach delivers superior accuracy for the same total bit count, particularly at lower bit numbers.

Given these findings, this study opts for the dynamic fixed-point method, setting each output at 18 bits. The two Ofmap_RAM storage units require storage for 8192 and 4096 values. Using 32-bit floating points for the output would demand 262,144 bits and 131,072 bits of memory, respectively. By selecting the 18-bit dynamic method, we achieve significant memory savings of 114,688 bits and 57,344 bits for the two Ofmap_RAM units.

The accuracy for each category is shown in Table 9, derived from a Python evaluation used to quantize the fixed-point networks. Notably, the second and eighth categories display lower accuracy levels. These categories correspond to 0.007-inch and 0.021-inch inner race faults, respectively. Their identification poses a challenge; for instance, the 0.007-inch inner race fault is often misclassified as a 0.021-inch inner race fault.

In total, there are 8680 weights. Originally, each weight is represented by 32 bits. After quantization, each weight requires only four bits, resulting in memory savings of 87.5%. The bias, Ofmap_RAM1, and Ofmap_RAM2—with 10, 8192, and 4096 values, respectively—transition from using 32-bit floating-point representations to 18-bit fixed-point numbers. This change means each value only needs 18 bits, saving 43.75% of memory space for these components. Additionally, the Input_RAM, comprising 4096 values, is optimized to use just nine bits per value, leading to a 71.88% reduction. Cumulatively, the overall memory savings is 63.49%, as shown in Table 10.

## 4. Experimental Results

When deploying on an FPGA, components such as Kernel_ROM, Input_RAM, Ofmap_RAM1, and Ofmap_RAM2 are constructed using the block memory generator, while the clocking wizard produces the system clock. Within the FPGA synthesis tool, the integrated logic analyzer (ILA) allows easy waveform visualization to facilitate debugging. Users can specify the signals and the depths they wish to observe via the ILA. However, it is essential to define the trigger conditions appropriately.

To achieve real-time capability, we analyze the requirement of the computing cycles and decide the clock rate. The CWRU dataset offers vibration signal samples at 12,000 and 48,000 samples per second. The circuit must wait for 4096 data points to be input before performing the CNN network operations using the 48,000 samples/second rate for illustration. An image containing 4096 time domain signal points for real-time state diagnosis must be processed within 0.085333 s. Completing the calculations for one such image requires 360,828 cycles, implying a required clock frequency of approximately 4.23 MHz for the intended hardware accelerator. However, generating a precise 4.23 MHz frequency during FPGA implementation is not feasible. Consequently, a 5 MHz clock frequency is employed in this work.

This research specifically utilizes the Xilinx Virtex-7 FPGA VC707 evaluation board to validate the circuit design. While the VC707 inherently offers a 200 MHz clock frequency, the necessary clock rate for our circuit is achieved using the clocking wizard. The circuit’s power consumption is 342 mW at a clock rate of 5 MHz. Notably, the circuit can function at a maximum clock rate of 140 MHz, which consumes 616 mW.

In the proposed design, a single Input_RAM is utilized to access the input signal. Once the initial convolution layer processes are finalized, the RAM values can be refreshed. With a frequency of 5 MHz, completing the first convolution layer requires 6574,500 ns, while generating the label output takes 72,165,700 ns. Vibration signals are fed every 20,833 ns. Hence, besides the 4096 values needed for the initial convolution layer’s computations, extra memory is necessary to accommodate the 316 vibration signals fed during this period. After the label generation, only 3464 vibration values are input, leading to a circuit idle time of 13,166,456 ns.

On the other hand, at 140 MHz clock rate, the first convolution layer completes in 236,682 ns, and producing the label output requires 2,597,965 ns. In this scenario, extra storage is required for the 12 vibration signals processed during the initial convolution phase. Post-label production, with just 125 vibration values input, the circuit remains inactive for 82,727,843 ns. The vibration signal’s sampling rate can be elevated, or the operational frequency can be adjusted to 4.23 MHz to optimize and prevent such inactivity. 

To assess real-time capabilities, we conduct a comparison between our method and a state-of-the-art real-time hardware solution [30] for bearing faults diagnosis. The referenced work [30] introduced a binary weight-based CNN on FPGA, achieving real-time performance at 130 MHz with 20,387,288 computing cycles and resulting in a detection iteration time of 156,819,019 ns. As previously noted, our solution, operating at a comparable clock rate (e.g., 140 MHz), demonstrates a significantly faster computational completion within 2,597,965 ns, thereby surpassing the performance of the referenced method.

Finally, In Table 11, the comparisons indicate that the proposed design, implemented with a lightweight network, has fewer parameters than other architectures. This reduction in parameters leads to decreased storage requirements. Regarding the FPGA implementation, LUT, DSP, FF, and BRAM usage in this work is significantly lower than in other comparable models, resulting in the lowest power consumption. The efficiency reported in this work stands at 1.126 (GOPS/W) when operating at 140 MHz.

## 5. Conclusions

This paper explores the design flow from software to hardware, applying various quantization approaches in the software to minimize both the quantity and the bit size of the model’s parameters. Based on the sampling rate provided by the CWRU dataset, the proposed design achieves real-time processing with a circuit frequency of 5 MHz. The proposed CNN hardware accelerator is implemented with an FPGA evaluation board (VC707). We process each image, containing 4096 values at a rate of 1.576 M samples per second, resulting in a processing time of 2,598,984 nanoseconds per image. However, at a circuit speed of 140 MHz, the processing time is reduced to just 2,597,965 nanoseconds without any loss in accuracy. This study performs quantization at four levels: 50%, 75%, 87.5%, and 100%, respectively, and rounds the parameters to the nearest power of two, facilitating operation without multiplication. We have effectively decreased memory usage by substituting floating points with fixed points of a lower bit width. The employed architecture is based on a lightweight network, totaling only 8.69 K parameters. For the implementation using VC707, when the clock frequency requirement is set to 140 MHz, it uses only 9689 LUTs, no DSPs, 5703 FFs, and 8.5 BRAMs, with a total power consumption of 616 mW. When the clock frequency requirement is set to 5 MHz, the resource usage slightly decreases to 9306 LUTs, with no DSPs, 5703 FFs, and 8.5 BRAMs, and the power consumption drops to 342 mW.

## Figures and Tables

**Table 1:** Label and corresponding bearing status.

Label	Fault Diameter	Bearing Status
0	No fault	health
1	0.007 inch	ball fault
2	0.007 inch	inner race fault
3	0.007 inch	outer race fault
4	0.014 inch	ball fault
5	0.014 inch	inner race fault
6	0.014 inch	outer race fault
7	0.021 inch	ball fault
8	0.021 inch	inner race fault
9	0.021 inch	outer race fault

**Table 2:** Number of training and test images for each category.

Label	Training Images	Validation Images	Test Images
0	259	54	100
1	401	120	125
2	398	115	133
3	1120	260	339
4	465	96	125
5	391	73	113
6	423	114	138
7	400	113	132
8	414	104	129
9	1046	280	326
Total	5317	1329	1660

**Table 3:** Different kernel sizes and the corresponding accuracy.

Kernel Size	Training (64%)	Validation (16%)	Test (20%)
1 × 3	92.41%	90.66%	92.34%
**1 × 5**	**98.70%**	**97.14%**	**97.16%**
2 × 2	95.35%	92.55%	94.09%
3 × 3	97.25%	94.65%	94.39%

**Table 4:** Different architectures and the corresponding accuracy.

Architecture	Output Filter				Parameter	Test
	Conv1	Conv2	Conv3	Conv4		
1	4	8	16	×	990	85.36%
2	8	16	32	×	3570	95.54%
3	16	32	64	×	13,530	97.34%
4	32	64	128	×	54,150	97.53%
5	4	8	16	16	2270	89.15%
**6**	**8**	**16**	**32**	**32**	**8690**	**97.16%**
7	16	32	64	64	34,010	97.28%
8	32	64	128	128	134,570	97.46%

**Table 5:** Comparison table of the software level.

	[24]	[25]	[27]	This Work
Algorithm	DNN	MSIQDE + DBN	2-D CNN	2-D CNN
Parameter (K)	1110	266	10,899	8.69
Category	7	10	10	10
Accuracy	100%	99.7%	99.77%	97.16%

**Table 6:** Accuracy with different quantization percentages.

	Training	Validation	Test
Unquantized	98.70%	97.14%	97.16%
Quantize 50%	97.98%	97.74%	95.78%
Quantize 75%	98.00%	97.81%	97.04%
Quantize 87.5%	98.00%	97.74%	96.80%
Quantize 100%	97.28%	95.93%	95.54%

**Table 7:** Cross-validation analysis after quantization.

Version	Training	Validation	Test
v1	97.28%	95.93%	95.54%
v2	92.56%	91.94%	92.46%
v3	97.91%	93.15%	93.85%
v4	98.45%	93.07%	93.43%
v5	96.40%	93.37%	92.77%
v6	96.00%	94.50%	92.95%
v7	98.56%	96.08%	95.48%
v8	94.86%	93.00%	94.75%
v9	98.23%	91.87%	93.73%
v10	95.33%	93.30%	91.38%

**Table 8:** Maximum and minimum values of each layer output.

Layer	Maximum	Minimum	Integer Bits
Conv1	13.078	−13.988	5
Conv2	41.105	−67.315	8
Conv3	273.757	−218.08	10
Conv4	1081.706	−1536.202	12
FC	437.778	−637.388	11

**Table 9:** Accuracy for each category.

Label	Test Image	Correct Image	Accuracy
0	100	100	100%
1	125	118	94.40%
2	133	110	82.71%
3	339	331	97.64%
4	125	112	89.60%
5	113	109	96.46%
6	138	137	99.28%
7	132	127	96.21%
8	129	115	89.15%
9	326	320	98.16%
Total	1660	1579	95.12%

**Table 10:** The memory usage of 32-bit floating point and this work.

	32-bit Floating Point	This Work	Reduction
Weight	277,760	34,720	87.5%
Bias	320	180	43.75%
Input ROM	131,072	36,864	71.88%
Ofmap RAM1	262,144	147,456	43.75%
Ofmap RAM2	131,072	73,728	43.75%
Total	802,368	292,948	63.49%

**Table 11:** Hardware accelerator comparison table.

	[31]	[32]	[33]	[34]	[35]	This Work
Technology	FPGA Altera Stratix V	FPGA VC709	FPGA ZYBO Z7	FPGA Zynq-7000	FPGA Zynq XC7Z045	FPGA VC707
Algorithm	F-CNN	CNN	CNN	CNN	CNN	CNN
Architecture	LeNet-5	LeNet	LeNet-5	LeNet-5	LeNet	LeNet
Dataset	MNIST	CIFAR10	MNIST	MNIST	MNIST	CWRU
Parameters (K)	430	N/A	13.47	61.5	33.6	8.69
Frequency (MHz)	150	100	100	150	N/A	5/140
Precision (bits)	32-bit	8-bit	32-bit	N/A	8-bit	18-bit
Power (W)	27.3	25.2	1.8	N/A	0.029	0.342/0.616
LUT	69,510	233,215	14,659	36,798	2800	9306/9689
DSP	23	2907	125	214	5	0/0
FF	87,580	307,617	14,172	N/A	2700	5703/5703
BRAM	510	477	119.5	123	7	8.5/8.5
GOPS	62.06	424.7	0.343	N/A	0.1	0.025/0.6938
GOPS/W	2.27	16.85	0.19	N/A	3.45	0.073/1.126
Accuracy	N/A	79.64%	N/A	98.4%	98.68%	95.12%

## Data Availability

Data are contained within the article.

## References

[1] Bellini A., Filippetti F., Tassoni C., Capolino G.-A. (2008). Advances in diagnostic techniques for induction machines. IEEE Trans. Ind. Electron..

[2] Motor Reliability Working Group (1985). Report of large motor reliability survey of industrial and commercial installations, Part I. IEEE Trans. Ind. Appl..

[3] Motor Reliability Working Group (1985). Report of large motor reliability survey of industrial and commercial installations, Part II. IEEE Trans. Ind. Appl..

[4] Motor Reliability Working Group (1987). Report of large motor reliability survey of industrial and commercial installations, Part III. IEEE Trans. Ind. Appl..

[5] Albrecht P.F., Appiarius J.C., McCoy R.M., Owen E.L., Sharma D.K. (1986). Assessment of the reliability of motors in utility applications—Updated. IEEE Power Eng. Rev..

[6] Li G., Li J., Fan H., Cao Y., Xu M., Wei J., Dong L. Model-based fault diagnosis method for gyro. Proceedings of the IEEE 3rd Information Technology, Networking, Electronic and Automation Control Conference (ITNEC).

[7] Liu T., Luo H., Yang Z. A novel data-driven fault classification method and its application to DC motor. Proceedings of the IEEE International Conference on Industrial Technology (ICIT).

[8] Niu G. (2017). Data-Driven Technology for Engineering System Health Management: Design Approach, Feature Construction, Fault Diagnosis, Prognostics, Fusion and Decisions.

[9] Case Western Reserve University (CWRU) Bearing Data Center Website. https://engineering.case.edu/bearingdatacenter.

[10] Liang M., Zhou K. (2021). Probabilistic bearing fault diagnosis using Gaussian process with tailored feature extraction. Int. J. Adv. Manuf. Technol..

[11] Mao W.T., Liu Y.M., Ding L., Li Y. (2019). Imbalanced fault diagnosis of rolling element bearing based on generative adversarial network: A comparative study. IEEE Access.

[12] Gao Y., Liu X., Xiang J. (2020). FEM simulation-based generative adversarial networks to detect bearing faults. IEEE Trans. Ind. Inform..

[13] Zhang W., Zhang P., He X., Zhang D. (2022). Convolutional neural network based two-layer transfer learning for bearing fault diagnosis. IEEE Access.

[14] Zhang R., Gu Y. (2022). A transfer learning framework with a one-dimensional deep subdomain adaptation network for bearing fault diagnosis under different working conditions. Sensors.

[15] Zhu M.H., Gupta S. (2017). To prune or not to prune: Exploring the efficacy of pruning for model compression. arXiv.

[16] Han S., Mao H., Dally W.J. (2016). Deep compression: Compressing deep neural networks with pruning trained quantization and Huffman coding. arXiv.

[17] Lin X., Zhao C., Pan W. Towards accurate binary convolutional neural network. Proceedings of the Conference on Neural Information Processing System (NIPS 2017).

[18] Li F., Zhang B., Liu B. (2016). Ternary weight networks. arXiv.

[19] Zhou A., Yao A., Guo Y., Xu L., Chen Y. (2017). Incremental network quantization: Towards lossless CNNs with low-precision weights. arXiv.

[20] Sánchez R.-V., Lucero P., Vásquez R.E., Cerrada M., Macancela J.-C., Cabrera D. (2018). Feature ranking for multi-fault diagnosis of rotating machinery by using random forest and KNN. J. Intell. Fuzzy Syst..

[21] FernáNdez-Francos D., Martínez-Rego D., Fontenla-Romero O., Alonso-Betanzos A. (2013). Automatic bearing fault diagnosis based on one-class v-SVM. Comput. Ind. Eng..

[22] Fadda M.L., Moussaoui A. (2018). Hybrid SOM–PCA method for modeling bearing faults detection and diagnosis. J. Braz. Soc. Mech. Sci. Eng..

[23] Al-Raheem K.F., Roy A., Ramachandran K.P., Harrison D.K., Grainger S. (2008). Application of the Laplace-wavelet combined with ANN for rolling bearing fault diagnosis. J. Vib. Acoust..

[24] Yang Y., Fu P., He Y. (2018). Bearing fault automatic classification based on deep learning. IEEE Access.

[25] Deng W., Liu H., Xu J., Zhao H., Song Y. (2020). An improved quantum-inspired differential evolution algorithm for deep belief network. IEEE Trans. Instrum. Meas..

[26] Xie Y., Zhang T. Feature extraction based on DWT and CNN for rotating machinery fault diagnosis. Proceedings of the 29th Chinese Control and Decision Conference (CCDC).

[27] Wen L., Li X., Gao L., Zhang Y. (2018). A new convolutional neural network-based data-driven fault diagnosis method. IEEE Trans. Ind. Electron..

[28] Iqbal M., Madan A.K. (2022). CNC machine-bearing fault detection based on convolutional neural network using vibration and acoustic signal. J. Vib. Eng. Technol..

[29] Li X., Yu S., Lei Y., Li N., Yang B. (2023). Intelligent machinery fault diagnosis with event-based camera. IEEE Trans. Ind. Inform..

[30] Chung C.-C., Liang Y.-P., Chang Y.-C., Chang C.-M. A binary weight convolutional neural network hardware accelerator for analysis faults of the CNC machinery on FPGA. Proceedings of the 2023 International VLSI Symposium on Technology, Systems and Applications (VLSI-TSA/VLSI-DAT).

[31] Zhao W., Fu H., Luk W., Yu T., Wang S., Feng B., Ma Y., Yang G. F-CNN: An FPGA-based framework for training convolutional neural networks. Proceedings of the Conference on Application-Specific Systems, Architectures and Processors (ASAP 2016).

[32] Liu Z., Dou Y., Jiang J., Xu J., Li S., Zhou Y., Xu Y. (2017). Throughput-optimized FPGA accelerator for deep convolutional neural networks. ACM Trans. Reconfigurable Technol. Syst..

[33] Dai R., Tang Y. Accelerator implementation of Lenet-5 convolution neural network based on FPGA with HLS. Proceedings of the Conference on Circuits, System and Simulation (ICCSS).

[34] Zhang L., Bu X., Li B. (2019). XNORCONV: CNNs accelerator implemented on FPGA using a hybrid CNNs structure and an inter-layer pipeline method. IET Image Process..

[35] Hailesellasie M.T., Hasan S.R. (2019). MulNet: A flexible CNN processor with higher resource utilization efficiency for constrained devices. IEEE Access.

